# Spontaneous Remission of Blastic Plasmacytoid Dendritic Cell Neoplasm: A Case Report

**DOI:** 10.3390/medicina60050807

**Published:** 2024-05-14

**Authors:** Tamara Castaño-Bonilla, Raquel Mata, Daniel Láinez-González, Raquel Gonzalo, Susana Castañón, Francisco Javier Díaz de la Pinta, Carlos Blas, José L. López-Lorenzo, Juan Manuel Alonso-Domínguez

**Affiliations:** 1 Hematology Department, Hospital Universitario Fundación Jiménez Díaz, 28040 Madrid, Spain; 2 Instituto de Investigación Sanitaria (IIS-FJD), Hospital Universitario Fundación Jiménez Díaz, 28040 Madrid, Spain; daniel.lainez@fjd.es; 3 Pathologycal Anatomy Department, Hospital Universitario Fundación Jiménez Díaz, 28040 Madrid, Spain

**Keywords:** blastic plasmacytoid dendritic cell neoplasm, spontaneous remission, acute myeloid leukaemia

## Abstract

Spontaneous remissions (SRs) in blastic plasmacytoid dendritic cell neoplasms (BPDCNs) are infrequent, poorly documented, and transient. We report a 40-year-old man presenting with bycitopenia and soft tissue infection. The bone marrow exhibited 3% abnormal cells. Immunophenotyping of these cells revealed the antigens CD45+ (dim), CD34+, CD117+, CD123+ (bright), HLA-DR+ (bimodal), CD56+ (bright), CD33+, CD13+, CD2+, and CD22+ (dim) and the partial expression of the CD10+, CD36+, and CD7+ antigens. All other myeloid, monocytic, and lymphoid antigens were negative. Genetic studies showed a complex karyotype and mutations in the TP53^R337C^ and KRAS^G12D^ genes. On hospital admission, the patient showed a subcutaneous nodule on the right hand and left lower limb. Flow cytometry multiparameter (FCM) analysis showed the presence of 29% abnormal cells with the previously described immunophenotype. The patient was diagnosed with BPDCN. The patient was treated with broad-spectrum antibiotics for soft tissue infection, which delayed therapy for BPDCN. No steroids or chemotherapeutic or hypomethylating agents were administered. His blood cell counts improved and skin lesions disappeared, until the patient relapsed five months after achieving spontaneous remission. About 60% of abnormal cells were identified. No changes in immunophenotype or the results of genetic studies were observed. The patient underwent a HyperCVAD chemotherapy regimen for six cycles. Consolidation therapy was performed via allogeneic bone marrow transplantation with an HLA-unrelated donor. One year after the bone marrow transplant, the patient died due to the progression of his underlying disease, coinciding with a respiratory infection caused by SARS-CoV-2. In the available literature, SRs are often linked to infections or other stimulators of the immune system, suggesting that powerful immune activation could play a role in controlling the leukemic clone. Nevertheless, the underlying mechanism of this phenomenon is not clearly understood. We hypothesize that the immune system would force the leukemic stem cell (LSC) to undergo a state of quiescence. This loss of replication causes the LSC progeny to die off, resulting in the SR of BPDCN.

## 1. Introduction

Blastic plasmacytoid dendritic cell neoplasm (BPDCN), previously known as agranular CD4+ CD56+ haematodermic neoplasm/tumour, is a rare and aggressive haematological malignancy derived from the plasmacytoid dendritic cell precursor (pDC) [1]. BPDCN represents fewer than 1% of haematological malignancies (the estimated annual incidence is 0.04 cases per 100,000 individuals in the USA). BPDCN can occur at any age; however, most patients are elderly, with a median age of presentation above 65 years [2]. Males tend to be affected slightly more than females, with some data showing an incidence ratio as high as 2.5:1. BPDCN does not appear to have any racial or geographic predisposition. Likewise, no environmental, inherited, or acquired genetic factors have been found to increase BPDCN risk.

Around 10–20% of patients with BPDCN have a previous diagnosis of acute myeloid leukaemia (AML), chronic myeloid leukaemia, chronic myelomonocytic leukaemia (CMML), or myelodysplastic syndromes [3,4,5,6,7]. The immunophenotypic profile is characterized by positivity for the CD56 and/or CD4 and CD123 markers and one other plasmacytoid dendritic cell (pDC) marker (*TCF4*, *TCL1*, CD303, or CD304) or the expression of any three pDC markers with negative results for other specific T-cell, B-cell, and myeloid cell markers [8]. In the 2016 and 2022 World Health Organization (WHO) classifications, BPDCN is included in the category of blastic plasmacytoid dendritic cell neoplasms and myeloid and histiocytic/dendritic neoplasms, respectively [9,10]. Nevertheless, BPDCN is not included in the International Consensus Classification (ICC) 2022 of myeloid neoplasms and acute leukaemia [11]. The most common form of presentation is skin involvement, but the disease can also manifest in the bone marrow (BM), peripheral blood (PB), lymph nodes, and central nervous system (CNS). Approximately 10–20% of BPDCN cases progress to a leukemic phase [2].

Prognosis is poor, with a median overall survival of 8 to 14 months, irrespective of the disease presentation pattern. No clinical, laboratory, or imaging findings have been associated with prognosis. Traditionally, the first line of treatment was remission induction therapy followed by a stem cell transplant (SCT) in eligible patients. Chemotherapy regimens developed for lymphoma, acute myeloid leukaemia, and acute lymphoid leukaemia have all been used to treat BPDCN. However, the goal of SCT is often not achieved with induction chemotherapy regimens. The CD123 antigen is universally expressed in BPDCN. This protein has a pathogenic role in the development of the disease, a finding which opened the door to the introduction of tagraxofusp, a recombinant human IL-3 bound to a truncated diphtheria toxin payload, as an initial treatment for BPDCN [12,13]. The term spontaneous remission (SR) in cancer refers to the recovery of a patient from cancer in the absence of a disease-specific treatment or in the presence of inadequate therapy. Therefore, neither chemotherapy nor radiotherapy should have been administered [14,15].

To date, there have only been five documented cases of spontaneous remission in BPDCN [16]. Here, we report one of the few cases described in the literature with a spontaneous complete remission of BPDCN, followed by relapse of the same disease within a short period of time.

## 2. Clinical Case

In April 2018, a 40-year-old man was evaluated for cellulitis on his right hand. Initially, no other pathological findings were observed upon physical examination. The complete blood count (CBC) showed a platelet count of 151 × 10^9^/L, a haemoglobin level of 7.9 g/L, and a white blood cell (WBC) count of 1.10 × 10^9^/L, with 26.3% neutrophils, 66.4% lymphocytes, 6.4% monocytes, and a normal proportion of basophils and eosinophils. Biochemistry tests noted normal levels of serum lactate dehydrogenase (LDH), at 336 U/L (normal range, 230–460 U/L), with elevated levels of acute-phase reactants such as ferritin (2.234.0 ng/mL; normal range, 20–250.0 ng/mL) and C-reactive protein (CRP) (43.7 mg/L; (normal range, 0–0.5 mg/L)). The remaining biochemical parameters were within normal ranges. Serological studies and urine and blood cultures were all negative. An ultrasound of the abdomen was normal.

**Figure 1:**
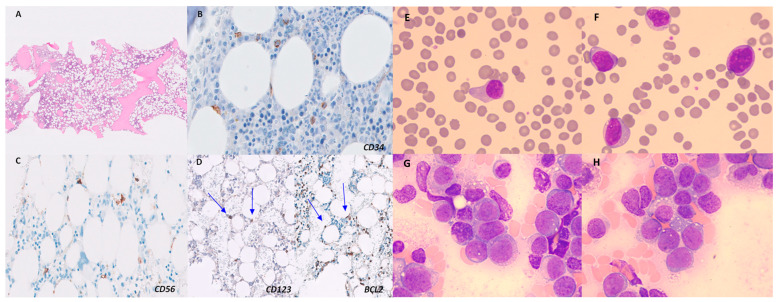
Biopsy specimen of bone marrow in April 2018. Scanning power (H&E, ×2) showed normocellular bone marrow without lymphoid aggregates (**A**). Higher-magnification scan (×40) showing large, scattered cells that were positive for CD123 and co-expressed BCL2, CD34, and CD56 (cells marked with blue arrows) (**B**–**D**). H&E = haematoxylin and eosin (**left image**). Blasts on peripheral blood smear (**E**,**F**) and bone marrow studies (**G**,**H**) (May–Giemsa staining, ×1000) in October 2018. We identified cells of medium size and regular nucleus with lax chromatin, without prominent nucleolus, and a large cytoplasm, slightly basophilic and agranular (**right image**).

The peripheral blood smear showed no immature cells. Bone marrow aspirate revealed 2% volume of blastic cells. In flow cytometry multiparameter analysis (FCM), an eight-colour combination of monoclonal antibodies was dropped over a bone marrow sample anti-coagulated with ethylenediamine tetra-acetic acid (EDTA), according to the manufacturer’s instructions. Later, erythrocytes were lysed using BD FACS™ Lysing Solution (BD Biosciences, Franklin Lakes, NJ, USA), and then two washing steps were performed with PBS (Inova Diagnostics, San Diego, CA, USA). Multi-colour FC antibody panels included the following: CD16 (FITC), CD13 (PE), CD34 (PerCP), CD117 (PE-Cy7), CD11b (APC), CD71 (APC-H7), CD64 (V450), CD45 (V500), HLA-DR (FITC, PerCP), CD123 (PE), CD36 (FITC), CD33 (PE, APC), CD14 (APC), CD56 (PE, PE-Cy7), CD15 (FITC), CD203c (PE), CD7 (APC), CD2 (PE-Cy7), CD5 (PerCP), CD4 (PE), CD8 (FITC), CD3 (V450), cyCD3 (V450), cyMPO (PE), CD19 (PerCP), CD20 (APC), CD22 (PE, APC), CD10 (PE, APC), CD38 (APC-H7), cyTdT (FITC), and cytoplasmatic immunoglobulin light chains (FITC and PE) (Table 1). An FACS Canto II cytometer (Becton Dickinson, Franklin Lakes, NJ, USA) was used to acquire 500,000 events per tube. Infinicyt™ software (Cytognos, Salamanca, Spain) was used to analyse the main leucocyte subpopulations in the generated FCS files. Immunophenotypic analysis showed 2.5% of abnormal cells that expressed the antigens CD45+ (dim), CD34+, CD117+, CD123+ (bright), HLA-DR+ (bimodal), CD56+ (bright), CD33+, CD13+, CD2+, and CD22+ (dim) and the partial expression of CD10+, CD36+, and CD7+. All other myeloid, monocytic, and lymphoid antigens were negative (CD3s/ic, CD5, TdT, CD79α, CD19, CD20, Kappa, Lambda, CD64, MPO, CD38, and CD203c). The histopathological bone marrow findings revealed normocellular bone marrow for the patient’s age, with scattered large blastoid cells located in the interstitum. No hypercellular aggregates were observed. Immunohistochemical analysis showed that large cells were positive for CD123 and co-expressed *BCL2*, CD34, and CD56. These cells accounted for 2–4% of the global cellularity in the core biopsy. The reticulin network was not increased (MF0) (Figure 1, left image). The bone marrow sample was processed after 24 h of unstimulated culture following standard procedures. G-banding staining was performed, and the karyotype was reported following the International System for Human Cytogenetic Nomenclature 2020 recommendations. The karyotype was complex (47, XY, −3, −5, of (7) (q21), 12, +13, of (20) (q12), + 3mar [3]/46, XY [5]). Next-generation sequencing (NGS) studies were performed using SOPHiA DDM™ Myeloid Solution (MYS, Sophia Genetics, Switzerland). This platform includes 30 relevant genes (*CEBPA*, *CSF3R*, *DNMT3A*, *ETV6*, *EZH2*, *JAK2*, *RUNX1*, *TET2*, *TP53*, and *ZRSR2*—which have complete coding sequences—and *ABL1*, *ASXL1*, *BRAF*, *CALR*, *CBL*, *FLT3*, *HRAS*, *IDH1*, *IDH2*, *KIT*, *KRAS*, *MPL*, *NPM1*, *NRAS*, *PTPN11*, *SETBP1*, *SF3B1*, *SRSF2*, *U2AF1*, and *WT1*). The platform performs an average of more than 99% of reads in target region with a depth of 1000× and a limit of detection of 2.5% for SNV and indels. NGS analysis showed the presence of *TP53^R337C^* and *KRAS^G12D^* mutations with variant allele frequencies (VAFs) of 5.2% and 4.8%, respectively.

After two days in hospital, subcutaneous nodules were detected on the left lower extremity and in the right hand. A histopathological study of the subcutaneous nodule on the left lower extremity did not reveal the presence of abnormal cells. An FCM study showed the presence of 29% abnormal cells with the previously described immunophenotype. Histological and flow cytometry studies on the subcutaneous nodule of the right hand showed no evidence of pathological cells. Based on these results, a diagnosis of BPDCN (immature blastic pDC neoplasm) was established [17].

**Figure 2:**
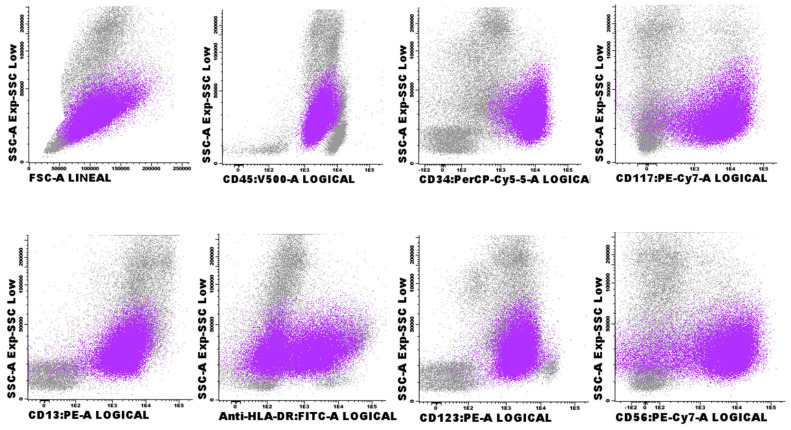
Flow cytometry of a bone marrow aspirate sample in October 2018 highlighting the abnormal pDCs population (purple). The rest of the haemopoietic cells of the bone marrow are shown in grey. Abbreviation: pDCs, plasmacytoid dendritic cells.

Induction chemotherapy was delayed on account of the patient’s active infection. Piperacillin–tazobactam was administered as intravenous antibiotherapy. A single dose of G-CSF was used to correct the neutropenia. In addition, the patient required the transfusion of two red blood cell concentrates. No steroids, chemotherapeutics, or hypomethylating agents were administered. Fifteen days after admission, both haemoglobin and leukocyte levels were gradually rising with the improvement in the patient’s clinical condition; in addition, skin lesions disappeared and the patient’s infection resolved. Serial follow-up blood tests were performed every 2–4 weeks with the normalization of the blood count in June 2018 (CBC showed a platelet count of 209 × 10^9^/L, a haemoglobin level of 13 g/L and a white blood cell count of 5.72 × 10^9^/L with 47% neutrophils, 39.7% lymphocytes, 6.5% monocytes, 5.9% eosinophils, and 0.9% basophils). A peripheral blood smear showed no immature cells. A bone marrow examination via FCM showed the disappearance of the clone (no cells with the same abnormal immunophenotype described at diagnosis were detected; limit of detection (LOD) of 0.1%), and the results of the cytogenetic (46 XY [20]) and NGS studies (no pathogenic variants were detected) were good. Subsequently, two analytical determinations were performed in July 2018 and August 2018. The values of the hemogram and the leukocyte formula were within the normal parameters. Eventually, in October 2018, the patient was admitted to hospital with a fever of 38 °C. Laboratory tests revealed thrombocytopaenia of 94 × 10^9^/L, a haemoglobin level of 9.4 g/dL, a white blood cell (WBC) count of 27.8 × 10^9^/L with 2% neutrophils, 11% lymphocytes, 3%, monocytes, 0% eosinophils, 2% basophils, and 82% blastic cells. The physical examination was normal. The peripheral blood smear and bone marrow aspirate showed 60% immature cells, respectively (Figure 1, right image). A biopsy was not performed on this sample. An FCM study detected the presence of 55% abnormal cells. The results of the immunophenotypic (Figure 2) and cytogenetic studies were superimposable to those evidenced in bone marrow at diagnosis. The NGS study revealed the same mutations detected at diagnosis with increased VAFs (*TP53^R337C^*, 38.8%; *KRAS^G12D^*, 22.10%).

**Figure 3:**
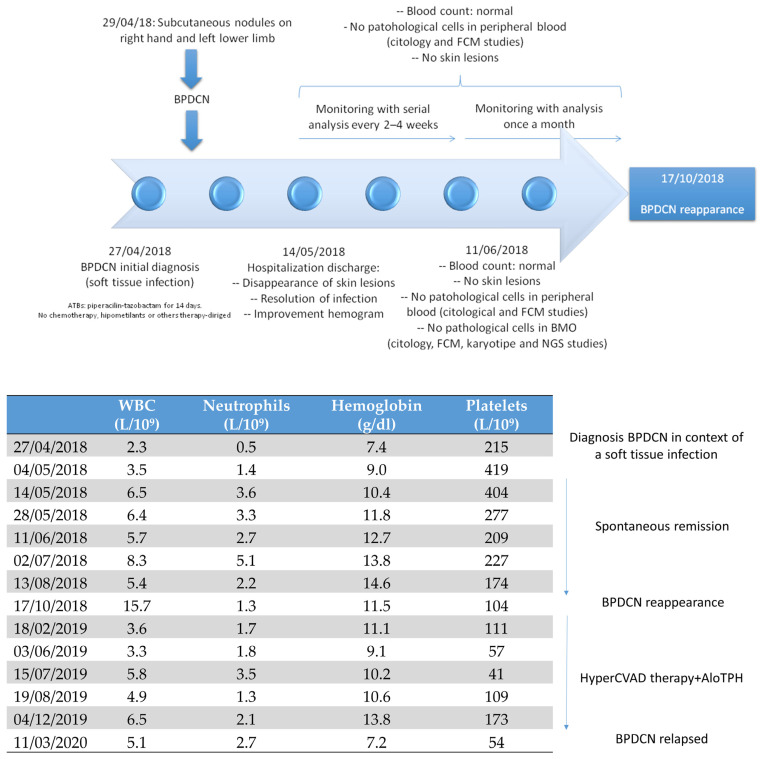
Natural history of our patient’s disease. Clinical and analytical evolution. Abbreviations: BPDCN, blastic plasmacytoid dendritic cell neoplasm; ATB, antibiotics; FCM, flow cytometry multiparametric; NGS, next-generation sequencing; BMO, bone marrow biopsy; WBC, white blood cell; AloTPH, allogeneic transplant progenitor hematopoietic.

The patient underwent a HyperCVAD chemotherapy regimen (cyclophosphamide, vincristine, doxorubicin, dexamethasone, methotrexate, and cytarabine) for six cycles and achieved a complete morphological remission (RCm) with negative measurable residual disease (MRD), as assessed via FCM. A total of four lumbar punctures were performed between November 2018 and February 2019, all of which were negative for malignancy. Consolidation therapy was performed via an allogeneic bone marrow transplantation with an HLA-unrelated donor conditioned with busulfan and fludarabine in April 2019. The patient reached complete chimerism, without graft-versus-host-disease data and controlled CMV replication up to the ninth month after transplantation. In March 2020, the patient was evaluated for fever and bycitopenia with the presence of 6% blasts in the peripheral blood smears. A bone marrow study showed 21% blasts. No changes were observed in the flow cytometry and genetic studies. The patient’s condition worsened clinically and haemodynamically. CT angiography of the abdomen was performed. Splenomegaly and adenopathic clusters were identified in relation to the progression of the underlying disease. Because of the epidemiological situation at that time, we performed a SARS-CoV-2 PCR and a chest X-ray examination and found bilateral pneumonia with SARS-CoV-2 positivity. The patient died ten days later as a consequence of a COVID-19-related respiratory infection (Figure 3).

## 3. Discussion

The SR of cancer has long been described in various malignancies. Neoplastic disease may partially or completely disappear without any treatment or as a result of a therapy considered inadequate to influence systemic neoplastic disease. The aetiology of SR has been connected to blood transfusions, infections, granulocyte-colony-stimulating factor therapy (G-CSF), the use of corticosteroids, and some biological subtypes of myeloid neoplasms. The two most well-known entities with frequent spontaneous remission are transient myeloproliferative disorder (TMD), often seen in newborns with Down syndrome, and AML with t(8;16)(p11;p13) in infants. While most TMD cases resolve spontaneously, 20–30% present with AML afterwards. SR has also been documented in MDS, particularly in children and young adults with abnormalities of chromosome 7 [14,18,19]. Although the precise mechanisms remain unclear, a possible mechanism through which severe infection may contribute to SR is through excessive and productive activation of proinflammatory cytokines, such as tumour necrosis factor (TNF) and interleukin-2 (IL-2), leading to increased activity of T-lymphocytes, macrophages and natural killer (NK) cells, all of which result in an anti-tumoral effect. In fact, previous case reports have shown elevated levels of TNF and IL-2, as well as increased NK cell activity, during active infections in patients exhibiting the SR of cancer [20].

Five cases of the SR of BPDCN have been documented so far. Although it is not possible to draw conclusions from this small cohort, the authors highlight the following points: (1) spontaneous remission in BPDCN may not be that uncommon; (2) in contrast to AML patients, those diagnosed with BPDCN with a normal karyotype are more likely to achieve spontaneous remission (although it is well known that 60% of patients diagnosed with BPDCN usually have a complex karyotype, as was the case with our patient); and (3) patients usually achieve at least partial remission in the skin, but similar bone marrow responses are unusual [16,21,22,23,24]. Here, we present a case of SR of BPDCN, probably one of the few cases related to tissue infection. Our patient was transfused with low numbers of blood products and received a single dose of G-CSF before remission. The role of both factors in the induction of remission cannot be completely excluded. The infectious disease was resolved after treatment with broad-spectrum antibiotherapy. Our patient achieved complete remission (CR) according to the criteria published in the international harmonization project on lymphoma (IHPL) [25] (Table 2).

A plausible mechanism of SR could be that any of the previously described triggers can produce the hyperactivation of the immune system against BPDCN stem cells. The immune system would then force the leukemic stem cells (LSCs) to undergo a state of quiescence. This loss of replication causes the LSC progeny to die off, resulting in the spontaneous remission of BPDCN. Therefore, regardless of the mechanism, LSCs do not re-enter the cell cycle, thus preventing the development of the disease.

Finally, our clinical case has several aspects that may generate controversy: (1) the diagnosis of BPDCN can be difficult to achieve, particularly when blast cells do not completely fit the typical immunophenotypic profile [26]. The case submitted may raise reasonable doubt between acute undifferentiated leukaemia and BPDCN. The intensity of the expression of some markers, such as CD123 and CD56, alongside the expression of other markers such as CD36, CD22, and CD10 would support the diagnosis of BPDCN. Additionally, CD36, CD10, and CD22 expression is very rare in undifferentiated AML [27,28]. Furthermore, the expression of the CD34, CD117, and CD33 markers is widely described in case series of patients diagnosed with BPDCN [29,30,31]. Lastly, while the CD13 marker is usually negative, some cases of CD13 expression in BPDCN have been reported [32]. Thus, the immunophenotyping data support the diagnosis of BPDCN [27,28]. (2) None of the diagnostic classifications of myeloid neoplasms (WHO 2016 and WHO/ICC 2022) describe leukaemia with maturation to dendritic cells as a differentiated entity [10,11,33]. However, some FCM expert groups have described series of patients diagnosed with blast plasmacytoid dendritic cells at different stages of maturation depending on their immunophenotypic profile [16,34]. Therefore, the phenotype of our patient may correspond to that of an immature/intermediate BPDCN. (3) The discrepancies in the results between the FCM and skin biopsy histopathologies could be explained by the higher diagnostic sensitivity of FCM or by the fact that the two laboratories received different samples, which could have influenced the magnitude of BPDCN infiltration. (4) Although the patient received antibiotics prior to disease spontaneous remission, the case meets the definition of spontaneous remission in cancer, which is defined as the recovery of a patient in the absence of disease-specific treatment or in the presence of inadequate therapy [35].

## 4. Conclusions

In summary, while some authors suggest that the spontaneous remission of BPDCN may not be as rare as previously thought, this assertion is based on just five reported cases of the SR of BPDCN (our case being the sixth). The SR of different haematologic neoplasms is transient, regardless of the unknown pathophysiology and triggering mechanism behind it. In future cases of SR, the characterization of the LSC cell cycle and the immune response could shed light on the mechanism behind SR and help us understand the pathophysiology of the regulation of LSC quiescence, which could open up new therapeutic pathways in haematological malignancies.

## Figures and Tables

**Table 1:** Markers used for flow-cytometry-based diagnosis of the blastic plasmacytoid dendritic cell neoplasm. Abbreviations: CD: clusters of differentiation; BD: Becton Dickinson; BC: Beckman Coulter.

Antibody and Fluorochrome	Clone	Manufacturer *
CD16-FITC	3GB	BD
CD2-PE-Cy7	L303	BD
CD5-PerCP	L17F12	BD
CD4-PE	13B8.2	BC
CD13-PE	L138	BD
CD34-PerCP	8g12	BD
CD117-PE-Cy7	104D1D2	BC
CD11b-APC	D12	BD
CD71-APC-H7	M-A712	BD
CD64-V450	10.1	BD
CD45-V500	2D1	BD
HLA-DR-FITC/PerCP	DK25//CR3/43	Palex//BD
CD123-PE	9F5	BD
CD36-FITC	FAG.152	BC
CD33-PE/APC	P67.6//P67.6	BD//BD
CD14-APC	M⊘P9	BD
CD56-PE/PE-Cy7	MY31//N901	BD//BC
CD15-FITC	MMA	BD
CD8-FITC	DK25	Palex
CD3-V450	UCHT1	BD
cyCD3-V450	UCHT1	BD
cyMPO-PE	_CLB_MPO	BC
CD19-PerCP	SJ25C1	BD
CD20-APC	L27	BD
CD22-PE/APC	S_HCL_1	BD
CD10-PE/APC	HI10a	BD
CD38-APC-H7	HB7	BD
cyTdT-FITC	HT-6	Palex
cyKappa-FITC	Polyclonal	Dako
cyLambda-PE	Polyclonal	Dako
CD7-APC	HIT7	Immunostep
CD203c-PE	NP4D6	BD

* Manufacturer: BD, Franklin Lakes, NJ, USA; BC, Brea, CA, USA; Palex, Barcelona, Spain; Dako Santa Clara, CA, USA; Immunostep, Barcelona, Spain.

**Table 2:** Reported cases of spontaneous remission of BPDCN.

Case Nº	Age/Sex	Sites of Involvement	Phenotype	Karyotipe	NGS	Response (Months)	Trigger Factor before SR	Second Treatment	Survival (mo)	References
1	87/M	Skin BM	CD123 CD56 TCL1 CD4	46,XY	NA	PR (2 m)	Sepsis	None	8 (dead)	Suzuki, A, et al., 2021 [16]
2	79/M	Skin	CD4 CD56	NA	NA	PR	NP	NA	NA	Pemmaraju, N, et al., 2019 [25]
3	78/M	Skin BM LN	CD123 CD56 TCL1 CD4	46,XY	NA	1st PR (2 m) 2nd PR (5 m)	ASR	THP-COP	18 (dead)	Daitoku S, et al., 2019 [22]
4	15/F	BM LN	CD4 CD56	46,XX	NA	PR	NA	Ara-C, VP-16, MIT	18 (alive)	Hashikawa K, et al., 2012 [24]
5	67/M	Skin Stomach	CD4 CD56 CD123	NE	NA	CR	NA	None (discharged)	NA	Yasuda H, et al., 2014 [23]
6	40/M	Skin BM	CD4 CD56 CD123	Complex karyotipe	*TP53 KRAS*	CR (5 m)	Soft tissue infection	HyperCVAD and aloTPH	18 (dead)	Present clinical case

Abbreviations: CR: complete remission; PR: partial remission; LN: lymph node; BM: bone marrow; NP: nothing particular; ASR: atraumatic splenic rupture; MIT: mitoxantrone; THP-COP: cyclophosphamide, pirarubicin, vincristine and prednisolone; NA: not available; NE: not evaluated.

## Data Availability

Data are available upon reasonable request from the authors after approval.
